# Comparison of rRNA-based and DNA-based nucleic acid amplifications for detection of *Chlamydia trachomatis*, *Neisseria gonorrhoeae*, and *Ureaplasma urealyticum* in urogenital swabs

**DOI:** 10.1186/s12879-018-3580-0

**Published:** 2018-12-12

**Authors:** Yuying Liang, Xin Jin, Fang Yuan, Zhanjia Li, Shuiping Chen

**Affiliations:** 1 Department of Infection Control, Affiliated Hospital of Academy of Military Medical Sciences, Beijing, People’s Republic of China; 2 Department of Laboratory Medicine, Affiliated Hospital of Academy of Military Medical Sciences, Beijing, People’s Republic of China

**Keywords:** *Chlamydia trachomatis*, *Neisseria gonorrhoeae*, *Ureaplasma urealyticum*, Simultaneous amplification and testing, qPCR

## Abstract

**Background:**

Nucleic acid amplification tests (NAAT) are well-accepted in diagnosis and surveillance of sexually infectious pathogens worldwide. However, performance differences between a RNA-based NAAT and DNA-based NAAT are rarely reported. This study compares the performances of the RNA-based SAT (simultaneous amplification and testing) assay and the DNA-based quantitative real-time polymerase chain reaction (qPCR) assay.

**Methods:**

A total of 123 urogenital swabs were collected from outpatients with suspected genital infections in our hospital. *Chlamydia trachomatis* (CT), *Neisseria gonorrhoeae* (NG), and *Ureaplasma urealyticum* (UU) in these swabs were simultaneously tested by SAT and qPCR. Any swabs were positive in the qPCR assay were further verified by following cloning and sequencing. All statistical analysis was performed using the SPSS software.

**Results:**

When the concentrations of CT, NG, or UU were more than 1 × 10^3^ copies/ml, 100% agreements between SAT and qPCR were observed regardless of the pathogen. No discrepancy was found. However, the sensitivity of SAT is significantly higher than qPCR in samples with concentration less than 1 × 10^3^ copies/ml. When tested by SAT and qPCR, 57.14 and 28.57% were positive for CT, 46.15% and 0 were positive for NG, 80% and 0 were positive for UU, respectively.

**Conclusions:**

The SAT assay has better agreements and higher sensitivities when compared with the qPCR assay, and thus could be a better choice for screening, diagnosis, and surveillance of sexually transmitted diseases, especially for CT and NG.

## Background

*Chlamydia trachomatis* (CT) and *Neisseria gonorrhoeae* (NG) are two of the most prevalent sexually infectious pathogenic bacteria worldwide with 131 million chlamydia infections and 78 million gonorrhea infections out of 357 million new infections per year (http://www.who.int/mediacentre/factsheets/fs110/en/). Although *Ureaplasma urealyticum* (UU) is considered as a commensal bacterium in the urogenital tract, it has been reported to be associated with some sexually transmitted diseases including non-gonococcal urethritis (NGU) [1, 2] and patients with high UU loads are associated with acute NGU [2]. Although these infections are curable, screening still remains very important to prevent late complications caused by these pathogens.

Traditionally, selective culture methods are used to detect CT, NG, and UU in clinical samples, but their sensitivities are very low [3, 4]. Nucleic acid amplification tests (NAAT), which have a better performance with high sensitivity, specificity, and ease of sample transport, have been approved by the Food and Drug Administration (FDA) for the detection of CT and NG, and widely used in clinical facilities to detect the sexually infectious pathogens [5, 6]. Recently, a novel RNA-based NAAT (simultaneous amplification and testing, SAT), which is based on transcription mediated amplification (TMA) and designed to detect pathogenic bacteria by amplification of 16S rRNA, has been reported to detect some sexual, respiratory, and enteric pathogens, including CT, NG, UU, *Mycoplasma genitalium, Mycobacterium tuberculosis*, *Mycoplasma pneumon*niae, entervirus 71, and coxsackievirus A16 [7–11]. SAT provides a great advantage because rRNA is about 10,000 times of the copy number of genomic DNA and 1000 times of that of plasmid DNA [12]. It is noteworthy that probes in SAT are labeled with fluorescences [8–11], while probes in another TMA-based detection method, APTIMA, are labeled with chemiluminescences [13]. Performance comparison of the RNA-based SAT with DNA-based NAAT assay is unclear yet.

Here, we compared the performances of the RNA-based SAT assay with the DNA-based quantitative real-time polymerase chain reaction (qPCR) assay by simultaneous detection of CT, NG, and UU in urogenital swabs.

## Methods

### Samples

A total of 123 urogenital swabs were collected from 123 outpatients with suspected genital infections in our hospital, which consisted of 64 male and 59 female with a median age of 29 years old (range: 19–63). Swabs were placed into 2 ml of sterile normal saline and the tubes were vortexed. For each sample, the two NAATs were performed at the same time. RNase-free tubes and tips were used in the SAT assay.

This study was exempted from a requirement for a statement of ethical approval from ethical committee of the affiliated hospital of academy of military medical sciences.

### DNA extraction and the qPCR assay

CT, NG, and UU qPCR kits (Liferiver Biotech) were used in the DNA-based NAAT. 1 ml of each sample was centrifuged at 13000 g for 5 min at room temperature. The supernatant was discarded and the pellet was washed twice with normal saline. After the second wash, the pellet was resuspended in 100 μl extraction buffer and heated at 100 °C for 10 min. After another centrifugation at 13000 g for 5 min, the supernatants were collected and used as DNA extracts.

The reaction mixtures were prepared as described by the manufacturer’s instructions. The amplifications were performed on a Roche LightCycler® 480 system under the following conditions: 94 °C 2 min, 40 cycles of 93 °C 15 s and 60 °C 60 s. If the qPCR assay was positive, the amplified segments were subjected to cloning and sequencing to confirm whether they were true or not. Both negative and positive controls were included in each run.

### The SAT assay

Briefly, 16S rRNA was isolated from samples by a capture oligomer via target capture by magnetic microparticles. The target rRNA was then reverse transcribed to generate cDNA fragments using Moloney murine leukemia virus reverse transcriptase and multiple RNA copies (100–1000) were produced from each cDNA copy by the T7 RNA polymerase. Afterwards, these RNA copies were transcribed into cDNA again and bound to fluorescence-labeled specific probes, which were labeled with 6-carboxyfluorescein (FAM) phosphoramidite at the 5′ end and with 4-[4-(dimethylamino) phenylazo] benzoic acid N-succinimidyl ester (DABCYL) at the 3′ end [10]. The SAT assay was performed according to the manufacturer’s instructions (Shanghai Rendu Biotechnology Co, Ltd). 400 μl of samples and 100 μl RNA nucleic acid extraction buffer were mixed in a 1.5 ml microcentrifuge tube and heated at 60 °C for 5 min. After incubation at room temperature for 10 min, RNA extraction was performed by magnetic beads. 30 μl of RNA extracts and 40 μl of amplification detection buffer were mixed as the final reaction mixture. After incubation at 60 °C for 10 min, the reaction mixture was immediately placed at 42 °C for 5 min. 10 μl of pre-heated (42 °C) enzyme reagent was then added. The tubes were immediately placed on a Roche LightCycler® 480 system and subjected to amplification with the following conditions: 40 cycles of 42 °C for 1 min. Both negative and positive controls were included in each run.

To evaluate the reproducibility of SAT, high (original) and low (100-fold diluted) concentrations of the positive controls in the kits (CT: 10^4^ and 10^2^ copies/μl; NG: 10^4^ and 10^2^ copies/μl; UU: 2 × 10^5^ and 2 × 10^3^ cfu/ml) were tested, respectively. Each control sample was tested five times.

### Statistical analysis

All statistical analysis was performed using the SPSS software package version 20.0. Frequencies were compared using Fisher’s exact test. *p* values less than 0.05 were considered statistically significant.

## Results

All swabs were firstly detected by the qPCR assay. Of the 123 urogenital swabs, 33 swabs were positive for CT, 32 swabs were positive for NG, and 29 swabs were positive for UU after confirmation by the qPCR assay and sequencing. All positive swabs had a concentration of more than 1 × 10^3^ copies/ml. The remaining 29 swabs were negative for CT, NG, and UU.

### Excellent agreements between the SAT assay and the qPCR assay

After 19 of the 33 CT-positive, 19 of the 32 NG-positive, 19 of the 29 UU-positive, and 29 pathogen-negative samples were tested by SAT, 100% agreements between the SAT assay and the qPCR assay were observed in samples with a concentration more than 1 × 10^3^ copies/ml, as shown in Table 1. Specifically, the sensitivity and specificity were 100% (19/19) and 100% (10/10) for CT, 100% (19/19) and 100% (10/10) for NG, and 100% (19/19) and 100% (10/10) for UU, respectively. No discrepancy was found. In addition, SAT exhibited a good reproducibility. Specifically, the coefficient of variations (CV) of CT, NG, and UU were 1.91, 2.94, and 1.25% for high concentration of positive controls, and 3.14, 0.28, and 2.28% for low concentration of positive controls, respectively.

**Table 1:** Comparison of CT, NG, and UU results in samples with concentration more than 1 × 10^3^ copies/ml

	SAT (+)	SAT (−)	Sensitivity (%)	Specificity (%)	Accuracy rate (%)
CT					
qPCR (+)	19	0	100	100	100
qPCR (−)	0	10			
NG					
qPCR (+)	19	0	100	100	100
qPCR (−)	0	10			
UU					
qPCR (+)	19	0	100	100	100
qPCR (−)	0	9			

### The SAT assay had higher sensitivity than the qPCR assay

Because the minimum quantification limits of CT, NG, and UU qPCR kits (Liferiver Biotech) were 1 × 10^3^ copies/ml, samples with concentrations less than the limit could not be accurately quantified or might be detected as negative. Thus, samples with concentrations less than 1 × 10^3^ copies/ml would be ideal to evaluate the sensitivities. However, due to less availability of this kind of samples, especially NG samples, diluted samples were used here. The remaining 14 of the 33 CT-positive, 13 of the 32 NG-positive, and 10 of the 29 UU-positive were diluted to 1 × 10^2^ copies/ml. Then, nucleic acids (DNA and RNA) of these diluted samples were simultaneously isolated and tested using both of the SAT assay and the qPCR assay.

Among the 14 CT samples, 28.57% (4/14) was positive in the qPCR assay, while 57.14% (8/14) was positive in the SAT assay (Table 2). Among the 13 NG samples, none was positive in the qPCR assay, while 46.15% (6/13) was positive in the SAT assay (Table 2). Among the 10 UU samples, none was positive in the qPCR assay, while 80% (8/10) was positive in the SAT assay (Table 2). These results indicated that the SAT assay had higher sensitivities than the qPCR assay in the detection of CT, NG, and UU.

**Table 2:** Comparison of CT, NG, and UU results in samples with concentration less than 1 × 10^3^ copies/ml

	SAT (+)	SAT (−)	Positive rate of SAT(%)	Positive rate of qPCR(%)	Accuracy rate (%)
CT					
qPCR (+)	4	0	57.14	28.57	71.43
qPCR (−)	4	6			
NG					
qPCR (+)	0	0	46.15	0	53.85
qPCR (−)	6	7			
UU					
qPCR (+)	0	0	80	0	20
qPCR (−)	8	2			

## Discussion

Although the emergence and spread of sexually infectious pathogens remains a major global public health concern, severe diseases caused by CT and NG are curable with antibiotics [14]. Routine screening and test of cure (TOC) after treatments are mainstays of disease control. However, sexually transmitted diseases are frequently asymptomatic and the local pathogen concentrations are too low, which might increase the difficulty of diagnosis. The SAT assay is designed to amplify 16S rRNA which has a great number of copies in cells [12]. Thus, the advantage of the SAT assay might be helpful when patients are asymptomatic or pathogen concentrations are very low.

In this study, the SAT assay shows excellent agreement and higher sensitivity when compared with the qPCR assay. The SAT assay detects all bacteria strains (CT, NG, or UU) which are positive by the qPCR assay and there are no discrepant results when direct detection of clinical samples is performed. In addition, the assay can detect pathogens with low concentrations (less than 1 × 10^3^ copies/ml) indicating a higher sensitivity. Our results are consistent with other studies where another rRNA-based assay (APTIMA, Hologic Inc) was compared with DNA-based assays [15–17].

TOC is recommended after initiation of treatment. Because traces of bacterial DNA can persist for prolonged periods after successful elimination of pathogens [18, 19] and only live metabolically active bacteria produce RNA, rRNA-based NAAT could potentially discriminate metabolically-active pathogens from dead ones so that RNA might be a better biomarker than DNA during surveillance of treatments. A previous study indicates that 95% of patients clear bacterial RNA at day 13 which is one day earlier than DNA [20]. These results suggest that rRNA-based NAAT could avoid potential overtreatment. Combination of its sensitivity and specificity, the SAT assay might be an ideal TOC tool during treatment.

Our results show the same results that rRNA-based NAATs appear to offer significantly higher sensitivity than DNA-based NAATs [15, 21]. Thus, current detections using DNA-based NAATs might underestimate the prevalence of sexually infectious pathogens and overestimated the effectiveness of anti-bacterial treatments.

Our study has three limitations. Firstly, it is performed at a single hospital which might limit the generalizability. Secondly, pathogen-positive samples with concentration less than 1 × 10^3^ copies/ml used in this study are diluted from clinical samples due to less availability, which might bias the results. Thirdly, the sample size is small and further large size samples are needed to better understand the performance of the SAT assay. Nevertheless, excellent agreement and higher sensitivity of the SAT assay are warranted in this study.

## Conclusions

This study demonstrates that the SAT assay has excellent agreements and higher sensitivities for CT, NG, and UU detection in urogenital swabs when compared with the qPCR assay. Thus, the SAT assay could be a better choice for screening, diagnosis, and surveillance of sexually transmitted diseases. However, considering high prevalence in healthy populations, UU screening is only recommended in symptomatic patients with suspected urogenital tract infections.

## Abbreviations

CT: *Chlamydia trachomatis*
NAAT: Nucleic acid amplification test
NG: *Neisseria gonorrhoeae*
qPCR: Quantitative real-time polymerase chain reaction
SAT: Simultaneous amplification and testing
UU: *Ureaplasma urealyticum*
